# Preoperative N-terminal pro-B-type natriuretic peptide concertation and prognosis of brain tumor patients: a 5-year follow up study

**DOI:** 10.1038/s41598-017-15394-6

**Published:** 2017-11-07

**Authors:** Adomas Bunevicius, Vytenis Deltuva, Edward R. Laws, Giorgio Iervasi, Arimantas Tamsauskas, Robertas Bunevicius

**Affiliations:** 1 Neuroscience Institute, Lithuanian University of Health Sciences, Kaunas, Lithuania; 2 Department of Neurosurgery, Brigham and Women’s Hospital, Boston, Massachusetts, USA; 3 CNR - Institute of Clinical Physiology, Pisa, Italy; 4 Academy of Medicine, Lithuanian University of Health Sciences, Kaunas, Lithuania

## Abstract

Increased N-terminal pro-B-type natriuretic peptide (NT-proBNP) concentration predicts poor prognosis of non-CNS cancer patients. We evaluated the association of NT-proBNP concentration with disease severity, discharge outcomes and prognosis of patients undergoing craniotomy for brain tumor. From January, 2010 until September, 2011 two-hundred and forty-five patients (age 55.05 ± 14.62 years) admitted for brain tumor surgery were evaluated for NT-proBNP serum concentration. Outcome at hospital discharge was evaluated with the Glasgow Outcome Scale (GOS). Most common diagnoses were meningioma (37%) and high-grade glioma (20%). Greater NT-proBNP concentration was associated with lower Barthel index (rho = −0.305, p = 0.001) and Mini Mental State Examination scores (rho = −0.314, p = 0.001) and with greater Hospital Anxiety and Depression scale Depression score (rho = 0.240, p = 0.026). Greater admission NT-proBNP concentration was associated with lower discharge GOS score after adjusting for patient age, gender and histological brain tumor diagnosis (β = −0.253, p < 0.001). Greater NT-proBNP concentration was also associated with greater 5-year mortality risk (HR = 1.845; 95%CI [1.166–2.920], p = 0.009) controlling for patient age, gender, history of cardiovascular disease, histological diagnosis and adjuvant therapy. In sum, greater pre-operative NT-proBNP concentration is associated with worse health status, unfavorable discharge outcome and shorter survival of brain tumor patients.

## Introduction

N-terminal pro-B-type natriuretic peptide (NT-proBNP) is produced by ventricular cardiomyocytes in response to volume or pressure overload^1^. It is an established biomarker for diagnosing and monitoring heart failure^2^. Natriuretic peptide receptors are present in the central nervous system where they modulate neuronal activity, and are important for normal brain functioning and neuronal recovery after injury^3,4^. Elevated serum NT-proBNP concentration is associated with impaired recovery and poor prognosis after stroke^5^, aneurysmal subarachnoid hemorrhage^6^ and traumatic brain injury^7^.

Serum NT-proBNP concentration is commonly elevated in cancer patients without overt cardiovascular disease^8,9^. Biological mechanisms underlying the increased production of NT-proBNP in cancer setting remain poorly understood, but it was demonstrated that greater concentration of NT-proBNP and other biomarkers of myocardial damage are associated with worse prognosis and shorter survival of cancer patients not receiving cardio-toxic chemotherapy^10^. These findings suggest that subclinical dysfunction of the cardiovascular system is common and has prognostic significance in cancer patients.

The prognosis of malignant gliomas remains dismal with median survival rarely exceeding 12 months despite of multimodal treatment modalities, while other, benign brain tumors, can be cured with gross total surgical resection^11^. Tumor grade, patient age and functional status, and extent of surgical tumor resection are the most important prognostic indicators for malignant brain tumors^12^. Two previous studies in primary and metastatic brain tumor patients reported that greater NT-proBNP concentration was associated with greater mass effect and extent of perifocal brain edema^13,14^. However, the association of NT-proBNP concentration with health status and prognosis of brain tumor patients was not reported.

Surgery is the most important diagnostic and treatment modality of brain tumor patients. Risk stratification for peri-operative complications after elective craniotomy is important for complication prevention, post-operative recovery and long-term prognosis^15^. Increase of preoperative serum concentration of NT-proBNP is a powerful and independent predictor for postoperative cardiovascular events and mortality in patients undergoing non-cardiac surgery^16^. However, there are no studies evaluating possible clinical value of pre-operative NT-proBNP assessment for peri-operative risk stratification of patients undergoing elective surgery for brain tumors.

In this prospective observational cohort study we aimed to evaluate the association of pre-operative NT-proBNP serum concentration with disease severity, discharge outcomes and prognosis of brain tumor patients undergoing elective craniotomy.

## Methods

### Patients

The study and its consent procedures were approved by the Bioethics Committee of the Lithuanian University of Health Sciences, Kaunas, Lithuania (No. P2-9/2003, date: 2010/12/10). All study procedures and methods were performed in accordance with the relevant guidelines and regulations. All patients gave signed informed consent prior to inclusion in the study.

Recruitment in the study took place between January, 2010 and September, 2011. Consecutive adult patients admitted for elective brain tumor surgery at the Department of Neurosurgery of Hospital of Lithuanian of Lithuanian University of Health Sciences, Kaunas, Lithuania were considered for this prospective observational cohort study. The study exclusion criteria covered inability to speak Lithuanian and cognitive impairment that prevented from completion of the study tasks.

### Study procedure

Patients were approached within 3 days of hospital admission and were evaluated for demographic characteristics (age and gender), clinical characteristics (histories of cardiovascular disease and prior brain tumor treatments), cognitive functioning (Mini Mental State Examination or MMSE^17^) and functional status (Barthel Index or BI^18^). During the same visit patients were administered the Hospital Anxiety and Depression scale (HADS)^19^ for evaluation of depressive/anxiety symptom severity. Extent of surgical resection of brain tumor was defined by reviewing operative reports and post-operative CT scans. The final histological diagnosis of brain tumor was obtained from the pathology reports. At discharge from the hospital outcome was evaluated using the Glasgow Outcome Scale (GOS)^20^. Information pertaining to adjuvant therapies (chemotherapy and/or radiotherapy) was obtained from medical records. Mortality data were collected from the national death registry.

### Instruments

Functional status was evaluated with the 10-item BI^18^ that evaluates daily functional dependence in dressing, bathing, feeding, grooming, transfers between bed and chair, bladder and bowel control, toilet use, mobility and climbing stairs. Global BI score ranges from 0 to 100 with lower scores indicating greater functional dependence.

Cognitive functioning was evaluated with the MMSE^17^. The total score on the MMSE ranges from 0 to 30, with higher scores indicating better cognitive functioning.

Depressive and anxiety symptom severity was evaluated using the 14-item HADS that evaluates depressive symptom (HADS-Depression) and anxiety symptom (HADS-Anxiety) severity during the previous two weeks before surgery with greater score indicating greater symptom severity^19^. The HADS is a well-validated instrument and is commonly used in neuro-oncology setting^21,22^.

### Discharge outcome

Discharge outcome was determined with the GOS^20^. Patients were considered to have unfavorable discharge outcomes if their GOS score ranged between 1 (death) and 3 (severe disability). The GOS has good inter-rater agreement and is commonly used for outcome assessment in neurosurgery.

### Mortality data

Five-year mortality data were collected from the national death registry by using the Lithuanian equivalent of a social security number. Deaths that occurred between the study entry date and November, 2015 were considered. No patients were lost to follow-up. Causes of death noted on the death certificate were coded according to the International Statistical Classification of Diseases and Related Health Problems, Tenth Revision, Australian Modification (ICD-10-AM).

### Blood assays

Venous blood samples were centrifuged, and serum samples were frozen at −40 °C and stored. Serum samples from all patients were analyzed in a single batch after completion of this study. Serum concentrations of NT-proBNP were assessed using a radioimmunoassay method (Roche cobas analyzer; Roche Diagnostics, UK), with normal serum concentrations of NTproBNP of < 157 ng/l^23,24^.

### Statistical analyses

Continuous data are presented as mean ± standard deviation and median [IQR], and categorical data as counts and percentages. NT-proBNP concentration values were not normally distributed; therefore, non-parametric statistical tests were used for median comparison and correlation analyses, and logarithmic transformations of NT-proBNP were performed for all regression models. For all tests, two-sided p values of < 0.05 were considered to indicate statistical significance.

First, we compared NT-proBNP concentration and proportion of patients with elevated NT-proBNP concentration as a function of histological brain tumor diagnosis and histories of cardiovascular disorders by using the Pearson x^2^ and Kruskall-Wallis tests. We also calculated Spearman correlation of NT-proBNP concentrations with patients’ age, and scores on the BI, HADS and MMSE.

Next, we evaluated whether NT-proBNP concentration on admission was associated with outcome at discharge by comparing GOS score as a function of elevated NT-proBNP concentration using the Kruskall-Wallis test. The association of NT-proBNP concertation and elevated NT-proBNP concentration (≥157 ng/L)with discharge GOS score and unfavorable discharge outcome (GOS score of ≤ 3), respectively, was investigated by performing univariate linear and binary logistic regression analyses, and adjusting for patients’ age, gender and histological brain tumor diagnosis. Receiver operating characteristic (ROC) curve analyses were also employed to assess the prognostic value of NT-proBNP concentration for poor discharge outcome.

Finally, we evaluated the association of NT-proBNP concentration above the median concentration value with overall survival by performing Kaplan-Meyer analyses. Cox regression analyses were used to evaluate the association of ln(NT-proBNP) concentration with 90-days and 5-year mortality risk adjusting for age (years), gender, histological brain tumor diagnosis, history of cardiovascular disease and adjuvant brain tumor treatment.

### Data availability

Available upon request.

## Results

Three-hundred and fifty-six patients were approached after the hospital admission and before surgery, and invited to participate in the study. Blood samples for evaluation of NT-proBNP serum concentrations were available from 245 (69%) patients (32% men and 68% women; mean age 55.05 ± 14.62 years). The proportion of patients with histories of cardiovascular disease was significantly lower in patients included in the study when compared to patients excluded from the analyses (11% vs. 19%, respectively, p = 0.03). Patient age (p = 0.36), brain tumor histological diagnosis (p = 0.9), psychiatric histories (p = 0.86), proportion of patients who underwent gross total tumor resection (p = 0.84), discharge outcome (p = 0.62) and 5-year survival rate (p = 0.42) were similar between excluded and studied patients. Cognitive functioning and depressive symptom severity was evaluated in 94 (38%) patients.

Clinical characteristics of the study patients are presented in Table 1. The patients were diagnosed with meningioma (37%), high-grade glioma (20%), pituitary adenoma (14%) and low-grade glioma (9%). Twenty seven (11%) and nine (4%) patients had histories of cardiovascular and psychiatric disorders, respectively. The majority of patients (88%) underwent gross total tumor resection.

**Table 1:** Demographic and clinical characteristics of the study patients (n = 245)

**Demographic and clinical characteristics**	**Mean ± SD and median [IQR] or count (percentage)**
Age	
Years	55.05 ± 14.62; 57.00 [22]
≥65 years	79 (32%)
Gender	
Men	79 (32%)
Women	166 (68%)
Histological diagnosis of brain tumor	
High-grade glioma	49 (20%)
Low-grade glioma	21 (9%)
Meningioma	91 (37%)
Pituitary adenoma	35 (14%)
Vestibular schwannoma	14 (6%)
Metastatic disease	11 (5%)
Other	23 (9%)
History of cardiovascular disease	27 (11%)
History of psychiatric disease	9 (4%)
Recurrent tumor	38 (16%)
Tumor resection	
Gross total	215 (88%)
Subtotal or biopsy	30 (12%)
Barthel index	
Total score	95.37 ± 13.44; 100.00 [5]
Score ≤ 90	14 (6%)
Hospital Anxiety and Depression scale	
Depression subscale (score)	4.78 ± 4.21; 4.00 [6]
Anxiety subscale (score)	6.38 ± 4.44; 6.00 [6]
Mini mental state examination, n = 94	
Total score	26.35 ± 4.21; 27.00 [5]
Score < 24	20 (8%)
Glasgow outcome scale at discharge	
Score	4.07 ± 0.94; 4.00 [1]
Unfavorable outcome	43 (18%)
Adjuvant therapy	
Radiotherapy or chemotherapy	70 (29%)
Radiotherapy	66 (27%)
Chemotherapy	25 (10%)
Survival time (months)	42.58 ± 23.14; 59.00 [45]
Mortality rate	
90 days	15 (6%)
5 years	91 (37%)

SD, standard deviation; IQR, interquartile range.

The median NT-proBNP concentration was 93.15 [173.00] ng/L and 80 (33%) patients had NT-proBNP serum concentrations above the laboratory reference value (Table 2). The median NT-proBNP concentration (X^2^ = 33.956, p < 0.001) and proportion of patients with elevated NT-proBNP concentrations (X^2^ = 13.096, p = 0.04) differed as a function of brain tumor histological diagnosis. Specifically, the median NT-proBNP concentration and proportion of patients with elevated NT-proBNP concentration were the greatest in patients with high-grade glioma, meningioma and metastatic brain tumors, relative to patients with low-grade glioma, vestibular schwannoma and meningioma. The NT-proBNP concentration was greater in high-grade relative to low-grade glioma patients (p = 0.001). The proportion of patients with elevated NT-proBNP concentration was greater in patients with cardiovascular disease histories relative to patients without cardiovascular disease histories (52% vs. 30%, respectively, p = 0.02). There was a strong and positive correlation between patient age and NT-pro-BNP concentration (rho = 0.581, p < 0.001).

**Table 2:** NT-proBNP concentration across histological brain tumor diagnoses.

NT-proBNP	All patients	Histological diagnosis						X^2^ (p)
		High-grade glioma	Low grade glioma	Meningioma	Pituitary adenoma	Vestibular schwannoma	Metastatic disease	
Concentration, ng/LMedian [IQR]	93.15 [173.00]	132.9 [146.20]	36.24 [59.24]	121.8 [194.80]	33.58 [106.07]	67.54 [94.67]	107.70 [426.82]	33.956 ( < 0.001)
Concertation ≥ 157 ng/L	80 (33%)^A^	17 (35%)	3 (14%)	39 (43%)	6 (17%)	3 (21%)	5 (46%)	13.096 (0.04)

^A^Numbers do not add up because patients with other histological diagnoses are not included in the table.

Greater NT-proBNP concentrations were associated with lower BI score (rho = −0.305, p = 0.001), lower MMSE score (rho = −0.314, p = 0.001) and greater HADS-Depression score (rho = 0.240, p = 0.026).

Greater NT-proBNP concentrations (X^2^ = 27.309 p < 0.001) and NT-proBNP concentration above the reference range (X^2 = ^10.913, p = 0.028) were associated with worse outcome at hospital discharge (Table 3). Greater admission NT-proBNP concentration was associated with lower discharge GOS score in univariate analyses (β = −0.348, p < 0.001) and after adjusting for patient age, gender and histological brain tumor diagnosis (β = −0.253, p < 0.001). NT-proBNP concentrations above the reference range were associated with greater odds for unfavorable discharge outcome in univariate analyses (HR = 2.529; 95%CI [1.292–4.950], p = 0.007) and after adjusting for patients’ age, gender and tumor histological diagnosis (HR = 2.268 95%CI [1.043.493], p = 0.039). Receiver operating characteristic analyses showed that pre-operative NT-proBNP concentration can accurately classify patients who are at risk for unfavorable outcome at hospital discharge (area under the ROC curve of 0.709, p < 0.001).

**Table 3:** The association of NT-proBNP concentrations with GOS score at discharge.

NT-proBNP concentration	Glasgow outcome scale (score)					X^2^ (p)
	1	2	3	4	5	
Median [IQR]	186.40 [956.02]	185.30 [5035.71]	153.70 [170.93]	98.35 [167.16]	61.28 [113.87]	27.309 ( < 0.001)
Concentration > 157 ng/L	5 (56%)	3 (60%)	14 (48%)	39 (34%)	19 (23%)	10.913 (0.028)

**Figure 1:**
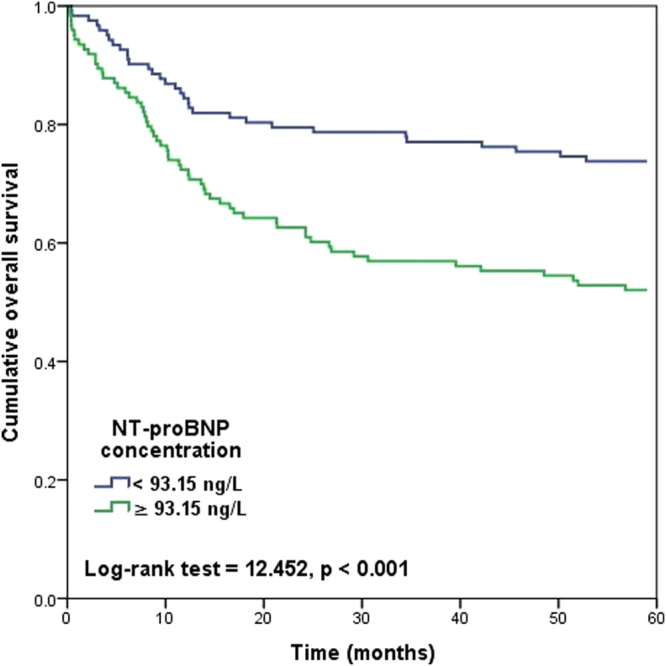
Kaplan-Meyer curve of 5-year survival as a function of NT-proBNP concentration above the median value.

Ninety-day and 5-year mortality rates were 6% and 37%, respectively. During the five year follow-up, there were 48 (98%) deaths in high-grade glioma patients; 2 (10%) in low-grade glioma patients; 20 (22%) in meningioma patients; 3 (9%) in pituitary adenoma patients; 2 (14%) in vestibular schwannoma patients; 11 (100%) in metastatic brain tumor patients; and 7 (5%) in patients with other histological diagnoses of brain tumors. The Kaplan-Meyer analyses showed that the 90-day mortality rate was significantly greater in patients with NT-proBNP concentration above the median value relative to patients with NT-proBNP concentration below the median value (10% vs. 3%, respectively, Log-rank = 5.654, p = 0.017). In Cox-regression analyses, greater NT-proBNP concentration was associated with greater odds for death within 90-days after the surgery in univariate analysis (HR = 3.085, 95%CI [1.385–6.874], p = 0.006) and adjusting for patient age, gender, history of cardiovascular disease and histological diagnosis (HR = 2.722, 95%CI [1.008–7.346, p = 0.048). The five year mortality rate was also significantly greater in patients with high vs. low NT-proBNP concentration (48% vs. 26%, Log-rank = 12.452, p < 0.001) (Fig. 1). Increasing NT-proBNP concentrations were associated with greater 5-year mortality risk in unadjusted analyses (HR = 1.985; 95%CI [1.400–2.814], p < 0.001) and after controlling for patient age, gender, history of cardiovascular disease, histological tumor diagnosis and adjuvant therapies (HR = 1.845; 95%CI [1.166–2.920], p = 0.009).

## Discussion

We found that elevated serum NT-proBNP concentrations were common to high-grade glioma, meningioma and metastatic brain tumor patients, and were associated with greater patient age and histories of cardiovascular disease. Elevated NT-proBNP concentrations were associated with worse functional health status, greater depressive symptom severity and worse cognitive functioning. Greater pre-operative NT-proBNP concentrations were associated with worse outcome at hospital discharge, and with greater 90-day and 5-year mortality risk independent of demographic characteristics and clinical disease severity.

One-third of the patients presented with elevated NT-proBNP concentrations. High-grade glioma, meningioma and metastatic brain tumor patient had the greatest NT-proBNP concentrations. Elevation of NT-proBNP levels in high-grade glioma and metastatic brain tumor patients can be attributed to more advanced tumor stage and correspond to previous findings in non-CNS cancer patients^9,10^. The increase of NT-proBNP concentrations in meningioma patients can be explained by advanced age, greater prevalence of cardiovascular histories and greater mass effect imposed by meningiomas that are usually benign and slow growing tumors.

The major output of NT-proBNP is from ventricular myocytes in response to pressure or volume overload, and that fact is therefore used to diagnose and monitor heart failure patients. However, biological mechanisms underlying NT-proBNP concentration elevation in cancer patients remain poorly understood^9,10^. For example, a very high NT-proBNP concentration was linked to fluid overload in hematologic malignancies^9^. On the other hand, another study in 99 cancer patients with multiple comorbidities and markedly elevated BNP values (>1000 pg/mL) did not find an association of BNP levels with volume overload or left ventricle dysfunction^8^. These findings suggest that in severely ill cancer patients elevation of NT-proBNP concentration cannot be explained solely by cardiac dysfunction, suggesting non-cardiac sources of NT-proBNP in cancer patients. Natriuretic peptides are produced by glioma cells and it was shown that BNP production is upregulated in astrocytoma cells in response to hypoxia^25^. Injury and hypoxia of non-malignant astrocytes in peri-tumoral areas can also increase BNP production and release. It is well documented that natriuretic peptides are present in the central nervous system^4,26^. Studies in traumatic brain injury patients found that more severe brain damage was associated with greater elevation of NT-proBNP concentrations in serum and in cerebrospinal fluid^7,27^. In a similar vein, greater mass effect and perifocal edema is associated with higher serum NT-proBNP concentrations in primary and metastatic brain tumor patients^13,14^. Necrosis and peri-tumoral edema are characteristics of rapidly growing aggressive brain tumors, and therefore can contribute to the observed increase of serum NT-proBNP concentrations. Further studies elucidating sources and mechanisms of increased NT-proBNP production and release in brain tumor patients are encouraged. Also, it remains to be studied whether NT-proBNP can directly modulate brain tumor aggressiveness and subsequently contribute to worse patient prognosis because natriuretic peptide receptors are present in glioma cells and modulate glioma gene expression^26^.

Greater NT-proBNP concentration was associated with shorter 5-year survival of brain tumor patients independently of age, gender, histories of cardiovascular disease, tumor grade, and adjuvant therapies, indicating that NT-proBNP can be a valuable prognostic biomarker above and beyond established clinical prognostic indicators. These findings agree with a previous study in 555 unselected cancer patients (including 23 patients with brain cancer) reporting that elevated NT-proBNP concentration was associated with shorter survival independent of patient age, tumor stage and entity, cardiac status, glomerular filtration rate and troponin T concentration^10^. The authors suggested that undiagnosed cardiac dysfunction in cancer patients can be an underlying mechanism of the observed association between elevated NT-proBNP concertation and greater mortality risk. A study in 2424 glioblastoma patients from the Swedish National Cancer Registry diagnosed between 1993 and 2006 found that glioblastoma patients were at 4-fold increased risk for incident heart failure relative to population-based controls^28^. Towards this end, our findings suggest that undiagnosed and/or subclinical dysfunction of the cardiovascular system is common in brain tumor patients, and is associated with poor prognosis. The clinical value of NT-proBNP measurement for detecting subclinical impairment of cardiovascular system of brain tumor patients should be addressed.

Elevated NT-proBNP concentration on admission was associated with worse outcome at hospital discharge and with greater 90-day mortality risk independent of age, gender and brain tumor histological diagnoses. These findings suggest that pre-operative NT-proBNP concentration can be a valuable biomarker for peri-operative risk stratification of patients undergoing elective craniotomy for brain tumor. It is well-documented that elevation of natriuretic peptide concentration is associated with greater complication risk and unfavorable outcomes of patients undergoing non-cardiac surgery^16,29^. A meta-analysis of 15 studies with 4856 patients undergoing non-cardiac surgery found that elevated preoperative NT-proBNP concentration was a strong and powerful predictor of all-cause mortality and major adverse cardiovascular events^29^. Another recent systematic review and meta-analysis of data from 2,179 patients undergoing non-cardiac surgery found that pre-operative natriuretic peptide was the strongest predictor of death and non-fatal myocardial infarction within 180 days after non-cardiac surgery^30^. Inclusion of natriuretic peptide measurement significantly improved the predictive power of clinical prognostic model for peri-operative complications. Therefore, it was recommended to consider NT-proBNP for perioperative risk stratification in patients undergoing non-cardiac surgery^31^. However, it should be noted that patients undergoing elective craniotomy were not included in the latter review studies. Our findings indicate that patients undergoing elective surgery for intracranial tumor and presenting with elevated NT-proBNP concentrations should be considered at greater risk for unfavorable discharge outcome and peri-operative mortality. Studies replicating our results and studying the additive prognostic role of natriuretic peptides for perioperative risk stratification in patients undergoing elective craniotomy for brain tumor are encouraged.

Greater NT-proBNP concentration was associated with greater functional impairment, worse cognitive functioning and more severe depressive symptoms. Previous studies showed that elevated NT-proBNP concentrations predict worse cognitive functioning and greater risk of cognitive decline in the general population^32,33^. An association of higher NT-proBNP concentration with greater depressive symptom severity was previously documented in patients with coronary disease^34^ and diabetes^35^. Cognitive decline and depression are common and distressing complication of brain tumor patients that are associated with worse prognosis^36–38^. Therefore, the possible clinical value of NT-proBNP assessment for initial diagnosis and prognosis of cognitive decline and mental distress in patients with brain tumors warrants additional research. Our findings suggest that NT-proBNP assessment should be considered in brain tumor patients presenting with cognitive impairment and depressive symptoms.

The study has limitations. Firstly, diagnoses of cardiovascular disorders were based on past medical histories and were not prospectively confirmed with cardiac ultrasound and/or other biomarkers of myocardial injury. Also, information about tumor size and perifocal edema was not recorded, preventing us from investigating an association between NT-proBNP concentration and tumor mass effect. Finally, our cohort included patients across a spectrum of brain tumors, and we were unable to evaluate the prognostic value of NT-proBNP in more homogenous patient subgroups. On the other hand, large sample size and long follow-up period are the major strengths of the study.

## Conclusions

Greater NT-proBNP concentration is associated with worse health status and with cognitive impairment of brain tumor patients. Elevated NT-proBNP concentration before surgery is associated with worse outcomes at hospital discharge and with worse prognosis of brain tumor patients. Therefore, NT-proBNP assessment can be considered for peri-operative risk stratification, prognostication and when evaluating cognitive/mental health status of brain tumor patients. Further studies investigating the clinical significance of NT-proBNP in brain tumor patients are recommended.
